# An extracellular polysaccharide is involved in the aluminum tolerance of *Pullulanibacillus* sp. CA42, a newly isolated strain from the Chinese water chestnut growing in an actual acid sulfate soil area in Vietnam

**DOI:** 10.3389/fmicb.2023.1241244

**Published:** 2023-08-28

**Authors:** Tomoko Aizawa, Junki Sato, Shimon Saito, Takanari Yasuda, Yutaro Maruyama, Makoto Urai

**Affiliations:** ^1^Department of Bioscience, College of Bioresource Sciences, Nihon University, Fujisawa, Kanagawa, Japan; ^2^Department of Applied Biological Sciences, College of Bioresource Sciences, Nihon University, Fujisawa, Kanagawa, Japan; ^3^Department of Chemistry for Life Sciences and Agriculture, Faculty of Life Sciences, Tokyo University of Agriculture, Tokyo, Japan

**Keywords:** *Pullulanibacillus*, extracellular polysaccharide, aluminum tolerance, plant-growth promoting rhizobacteria, structure, NMR spectroscopy

## Abstract

A novel aluminum-tolerant bacterial strain CA42 was isolated from the aquatic plant *Eleocharis dulcis*, which grows in a highly acidic swamp in Vietnam. Inoculation with CA42 allowed *Oryza sativa* to grow in the presence of 300 μM AlCl_3_ at pH 3.5, and biofilms were observed around the roots. Using 16S rRNA gene sequencing analysis, the strain was identified as *Pullulanibacillus* sp. CA42. This strain secreted large amounts of an extracellular polysaccharide (CA42 EPS). Results from structural analyses on CA42 EPS, namely methylation analysis and nuclear magnetic resonance (NMR), indicated that the chemical structure of CA42 EPS was a glycogen-like α-glucan. Purified CA42 EPS and the commercially available oyster glycogen adsorbed aluminum ions up to 15–30 μmol/g dry weight. Digestion treatments with α-amylase and pullulanase completely attenuated the aluminum ion-adsorbing activity of purified CA42 EPS and oyster glycogen, suggesting that the glycogen-like structure adsorbed aluminum ions and that its branching structure played an important role in its aluminum adsorbing activity. Furthermore, the aluminum tolerance of CA42 cells was attenuated by pullulanase treatment directly on the live CA42 cells. These results suggest that CA42 EPS adsorbs aluminum ions and is involved in the aluminum tolerance mechanism of *Pullulanibacillus* sp. CA42. Thus, this strain may be a potential plant growth-promoting bacterium in acidic soils. In addition, this study is the first to report a glycogen-like polysaccharide that adsorbs aluminum ions.

## 1. Introduction

Acid sulfate soil is the common name given to soils containing iron sulfides (pyrites). It is estimated that this kind of soil covers approximately 500,000 km^2^ worldwide (Michael et al., 2017) and is especially abundant in Australia and Asia. When acid sulfate soil is dug up, pyrite becomes exposed to the air and is oxidized to sulfuric acid; consequently, both soil and water show a lower pH. Such soil is known as actual acid sulfate soil (AASS) (Dent and Pons, 1995). Below pH 4.5, aluminum becomes more soluble and toxic to plants, and a few micronutrients, such as manganese (Lasat, 2002) and iron (McGrath et al., 2001), become more soluble and toxic. Most plant nutrients, especially phosphorus, become more limited in acidic soils (Kochian et al., 2004), leading to serious environmental destruction and significant economic problems.

However, few plants can survive in such extreme environments. Since it has become clear that acid- and aluminum–resistant microorganisms coexist in the rhizosphere of such plants, such microorganisms may be used for AASS bioremediation and crop cultivation. The use of these microorganisms in protecting the microenvironment of the plant surface and rhizosphere may yield a new and effective bioremediation method that can be used to recover AASS. Furthermore, protecting only the plant surfaces and rhizosphere is economical and has a minimal environmental impact.

In our previous study on developing bioremediation measures for AASS, we isolated several bacteria associated with plants grown in the highly acidic aquatic environments of AASS in Southeast Asia and reported new bacterial species (Aizawa et al., 2007, 2008, 2010a,b). For instance, we isolated a new strain of *Acidocella aluminiidurans* AL46 from *Panicum repens* grown in a highly acidic swamp in the AASS of Vietnam (Kimoto et al., 2010). AL46 grows at pH 3.0 and is tolerant to 500 mM aluminum sulfate or 200 mM aluminum chloride, which are higher than those observed in other bacteria. Furthermore, AL46 produces an aluminum-binding capsular polysaccharide that may be involved in resistance to high concentrations of aluminum (Aizawa and Urai, 2020). An aluminum-tolerant bacterium, CA42, was recently isolated from the Chinese water chestnut *Eleocharis dulcis*, growing in a highly acidic swamp in the AASS of Vietnam. CA42 was selected for plant growth promotion in acidic soils based on its ability to restore rice growth in inoculation experiments in the presence of aluminum ions. CA42 also produces a large amount of extracellular polysaccharide (EPS).

In the present study, we identified and characterized CA42 and determined the structure and aluminum-adsorbing ability of the EPS it produces. Furthermore, we validated the involvement of EPS in the aluminum tolerance of CA42.

## 2. Materials and methods

### 2.1. Isolation of the aluminum-tolerant strain CA42

CA42 was isolated from *E. dulcis* by using diluted tryptic soy broth 1:10 (1/10 TS) agar plates, which were prepared by diluting 2.75 g tryptic soy without glucose broth (BD, Franklin Lakes, NJ, United States) per liter of water and solidified with 15.0 g of agar (BD) per liter (pH 4.0). Acidic plates were prepared by mixing double-strength TS medium component without agar and 3% agar solution that had been autoclaved separately to prevent agar hydrolysis. The growth of CA42 in the presence of AlCl_3_ was tested using modified TS (MTS) medium (2.75 g·l^−1^ tryptic soy without glucose, 0.2 g·l^−1^ MgCl_2_·7H_2_O, 0.1 g·l^−1^ CaCl_2_·2H_2_O, 0.1 g·l^−1^ NaCl, 0.02 g·l^−1^ FeCl_2_·6H_2_O, 0.5 g·l^−1^ (NH_4_)_2_SO_4_; pH 4.0).

Furthermore, the AlCl_3_ tolerance of CA42 was examined through a growth promotion test (inoculation experiments) on rice plants (IR36) in Yoshida’s solution (pH 3.5) containing 300 μM AlCl_3_. Briefly, IR36 seeds were surface-sterilized as described by Kochian and Shaff (1991). Inoculation experiments were performed as described by Elbeltagy et al. (2001). Inoculated plants were cultivated in Yoshida’s solution (pH 3.2) (Yoshida, 1976) for 60 days in growth chambers (LPH-350S; Nippon Medical & Chemical Instruments Co. Ltd., Osaka, Japan) with 100 μmol m^−2^ s^−1^ illumination from cool-white fluorescent lamps at a 12-h photoperiod at 30°C.

### 2.2. Characterization of CA42

The 16S rRNA gene of strain CA42 was amplified via PCR using universal primers (Tamura et al., 2001), and the nearly complete 16S rRNA gene nucleotide sequence (1,546 bp) was obtained. Sequence similarity was determined using the featured identification service of EzBioCloud (16S-based ID)^1^ (Yoon et al., 2017). Multiple alignments of the sequence data were performed using ClustalX (Thompson et al., 1997). Subsequently, phylogenetic relationships with closely related species were determined using MEGA version 11 (Tamura et al., 2021). Evolutionary distances were computed as previously described (Jukes and Cantor, 1969). Phylogenetic trees were constructed using the maximum parsimony (Kluge and Farris, 1969), maximum likelihood (Felsenstein, 1981), and neighbor-joining (Saitou and Nei, 1987) methods. The reliabilities of these tree topologies were evaluated using bootstrap analysis with 1,000 replicates (Felsenstein, 1985).

The production of indole acetic acid was tested by using 1/10 TS containing 1% (w/v) glucose and 5 mM tryptophan (pH 3.5 and 6.0).

### 2.3. Extraction and purification of CA42 EPS

CA42 EPS was extracted from cells cultivated on 1/10 TS agar plates and purified using enzymatic treatments, phenol-chloroform treatment, and ethanol precipitation, as previously described (Urai et al., 2006).

### 2.4. Monosaccharide analysis

CA42 EPS (50 μg) was completely hydrolyzed using 2 M trifluoroacetic acid (TFA) at 100°C for 3 h. The obtained monosaccharides were labeled with 4-aminobenzoic acid ethyl ester (ABEE) and analyzed using high-performance liquid chromatography (HPLC; Nexera System; Shimadzu, Kyoto, Japan), as described previously (Urai et al., 2006). To determine the absolute configuration of CA42 EPS, the TFA hydrolysate of EPS was converted into acetylated (−)-2-butyl glycoside and analyzed using gas–liquid chromatography (GLC) (Leontein et al., 1978; Gerwig et al., 1979).

### 2.5. Nuclear magnetic resonance (NMR) analysis

NMR spectra were recorded at 500 MHz (^1^H) and 125 MHz (^13^C) using an ECA 500 instrument (JEOL Ltd., Tokyo, Japan) or at 600 MHz (^1^H) and 150 MHz (^13^C) with an ECZ 600 instrument (JEOL Ltd.). Chemical shifts were administered in parts per million (ppm), with acetone (δ ^1^H 2.23 ppm, δ ^13^C 31.1 ppm) used as an internal reference for samples measured in D_2_O solutions. Signals were assigned based on the results of the heteronuclear single-quantum coherence (HMQC) and heteronuclear multiple-bond coherence (HMBC) experiments. ^1^H NMR chemical shifts of the overlapping signals were obtained from the center of the cross peaks in the 2D spectra.

### 2.6. Methylation of CA42 EPS

Methylation of CA42 EPS was performed as described previously (Ciucanu and Kerek, 1984). Subsequently, the methylated polysaccharide was hydrolyzed, reduced with sodium borodeuteride, acetylated, and analyzed using GLC–mass spectrometry (MS) (GCMS-QP2020NX; Shimadzu, Kyoto, Japan).

### 2.7. Enzymatic digestion of CA42 EPS

CA42 EPS was digested with pullulanase (Sigma-Aldrich Co. LLC, St. Louis, MO, United States) or α-amylase (Tokyo Chemical Industry Co., Ltd., Tokyo, Japan) in 0.1 M acetate buffer (pH 5.0) at 37°C overnight. After denaturation of the enzyme via heat treatment at 100°C for 5 min, the products were recovered via freeze-drying. Oyster glycogen (FUJIFILM Wako Pure Chemical Corporation, Tokyo, Japan) and amylose (Sigma-Aldrich Co. LLC) were added using the same procedure.

Gel filtration column chromatography was performed using the Bio-Gel P-2 gel filtration column (900 mm × 15 mm ϕ; Bio-Rad Laboratories, Inc., CA, United States), with 0.2 M acetic acid as the eluent. Fractions containing saccharides were monitored using the phenol-H_2_SO_4_ method (Dubois et al., 1956).

### 2.8. Evaluation of aluminum ion-adsorbing activity of CA42 EPS

The aluminum ion-adsorbing ability of CA42 was measured according to methods reported by Kerven et al. (1989) and Maejima et al. (2017). A colorimetric method using the pyrocatechol violet (PCV) reagent was used to measure the free aluminum ions in the glucan samples containing the aluminum solution. An aqueous solution of CA42 EPS or standard glucan (final concentration of 1 mg/mL) was mixed with AlCl_3_ to a final concentration of 25 μM, and the concentration of free aluminum ions was measured by determining the adsorbance of PCV at 585 nm. The reduction in free Al^3+^ ions observed in the presence of glucans was considered as the amount of Al^3+^ adsorbed by the glucan.

### 2.9. Effect of pullulanase treatment on the aluminum tolerance of CA42

CA42 was cultured in 1/10 TS broth at 28°C for 2 d, and cells were recovered using centrifugation at 10,000 × *g* for 10 min. Cells were resuspended in 1 mL of 0.1 M acetate buffer (pH 5.0). Then, 30 μL of pullulanase was added, and the cells were incubated at 37°C for 4 h. Pullulanase-treated cells were recovered using centrifugation at 10,000 × *g* for 10 min, and the cells were rinsed with acetate buffer. Then, 5 μL of 1 M AlCl_3_ solution was added, and the cells were incubated at 28°C for 4 h. The cells exposed to aluminum ions were rinsed with 0.85% (w/v) saline, while the number of living cells was measured by calculating the number of colony-forming units on the 1/10 TS agar plates. *Escherichia coli* BW25113 was used as a control to evaluate aluminum toxicity. The survival rate of each strain was compared to that of cells that were not exposed to aluminum ions.

### 2.10. Statistical analysis

SPSS software (version 20.0; IBM Japan, Tokyo, Japan) was used. Normality of distribution and equality of variance were assessed using the Shapiro–Wilk’ and Levene’s tests. *p* values <0.01 were considered significant.

## 3. Results

### 3.1. Isolation and characterization of the aluminum-tolerant strain CA42

**Figure 1:**
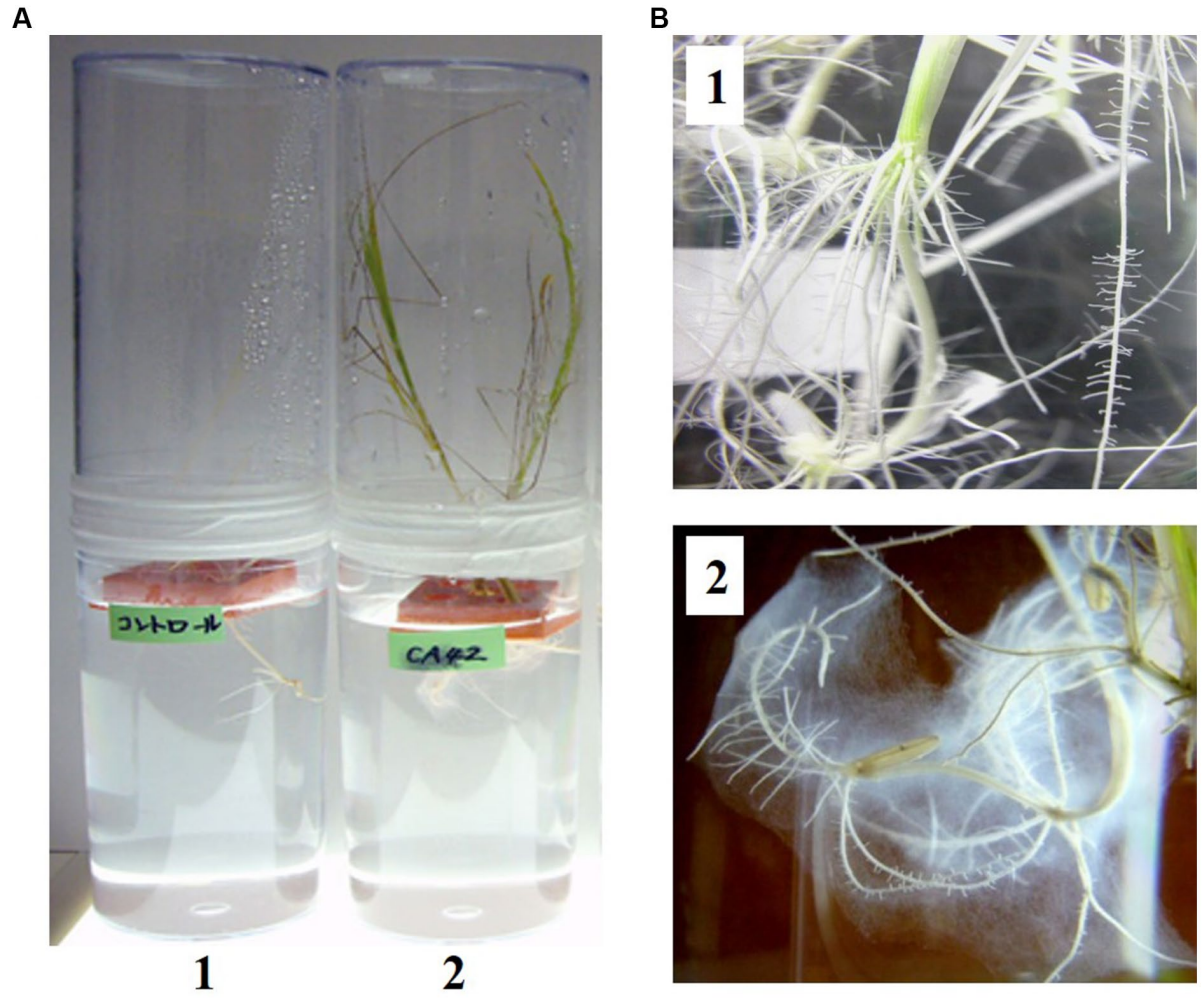
Inoculation of *Oryza sativa* cultivar *indica* IR36 with *Pullulanibacillus* sp. CA42 in Yoshida’s solution (pH 3.5) containing 300 μM aluminum chloride **(A)**. 1, Non-inoculated rice. 2, Rice inoculated with *Pullulanibacillus* sp. CA42. Biofilm around the roots of the rice inoculated with *Pullulanibacillus* sp. CA42 was observed. Enlarged photo of each root **(B)**.

Among the bacteria isolated from *E. dulcis* using 1/10 TS agar plates containing 5 mM AlCl_3_ (pH 3.5), strain CA42 restored the growth of rice plants in Yoshida’s solution (pH 3.5) containing 300 μM AlCl_3_ (Figure 1A). In addition, biofilm was observed around the roots of the rice plants inoculated with CA42 (Figure 1B). The organism formed yellowish, round, smooth, and flat colonies with entire margins. The strain showed good growth on 1/10 TS agar plates at 20–37°C, with optimal growth at 30°C. Strain CA42 showed good growth at pH 4–6, with optimal growth at pH 5 when cultured at 30°C for 5 d.

**Figure 2:**
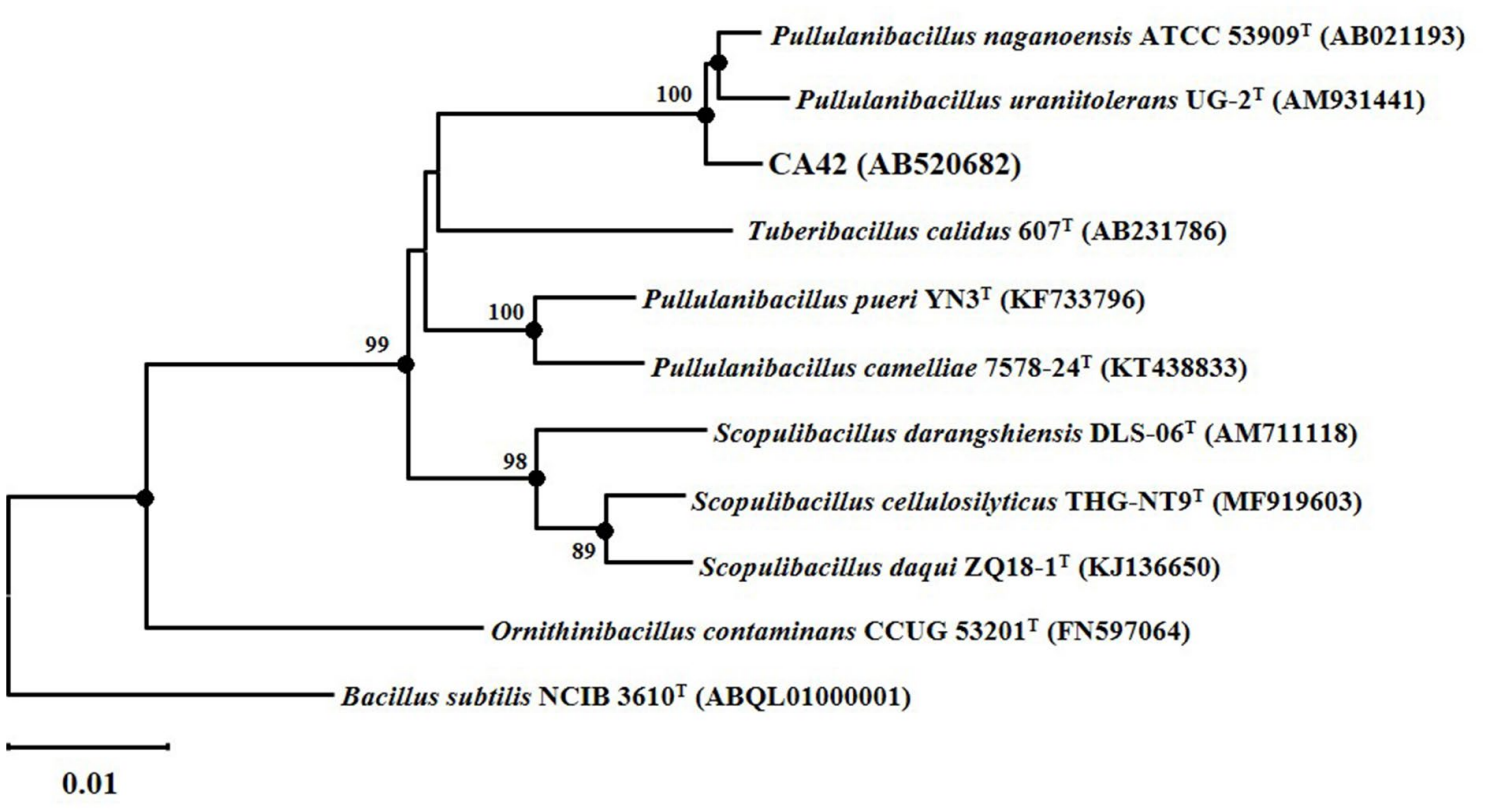
Neighbor-joining tree based on a nearly complete 16S rRNA gene sequence, showing the positions of strain CA42 and its phylogenetic neighbors. Filled circles indicate the corresponding nodes (groupings) that were also recovered in the maximum-likelihood and maximum-parsimony trees. Numbers at the nodes are percentages of bootstrap values based on 1,000 resampled datasets; only those above 70% are indicated. The sequence of *Bacillus subtilis* NCIB 3610^T^ was used as an outgroup. Bar, 0.01 nucleotide substitutions per nucleotide position.

The 16S rRNA gene sequence of CA42 showed 98.38 and 97.95% similarity to those of *P. naganoensis* (AB021193) and *P. uraniitolerans* (AM931441), respectively. The partial 16S rRNA gene sequence of CA42 was submitted to the GenBank/EMBL/DDBJ databases (Accession number: AB520692). Furthermore, phylogenetic analysis results revealed that strain CA42 belonged to the genus *Pullulanibacillus* (Figure 2). These and other physiological and biochemical data (unpublished) suggest that the isolate represents a novel species of the genus *Pullulanibacillus*.

CA42 produced indole acetic acid in tryptophan-containing medium, indicating that CA42 belongs to plant-growth promoting rhizobacteria. Furthermore, CA42 showed good growth in the presence of 5 mM AlCl_3_ in MTS medium and produced a large amount of EPS on 1/10 TS agar plates.

### 3.2. CA42 produced a glycogen-like EPS

Purified CA42 EPS was observed to be a white fibrous compound soluble in hot water and dimethyl sulfoxide, but not in cold water, methanol, or ethanol. At 280 or 255 nm, little or no adsorption was detected, suggesting that the CA42 EPS did not contain proteins or nucleic acids. Furthermore, the monosaccharide content of CA42 EPS was determined via TFA hydrolysis, followed by HPLC analysis. The results showed the presence of glucose as the sole polysaccharide component. The absolute configuration of glucose was determined using GLC with the acetylated (−)-2-butyl derivatives. The results showed that the glucose had the d-configuration.

**Figure 3:**
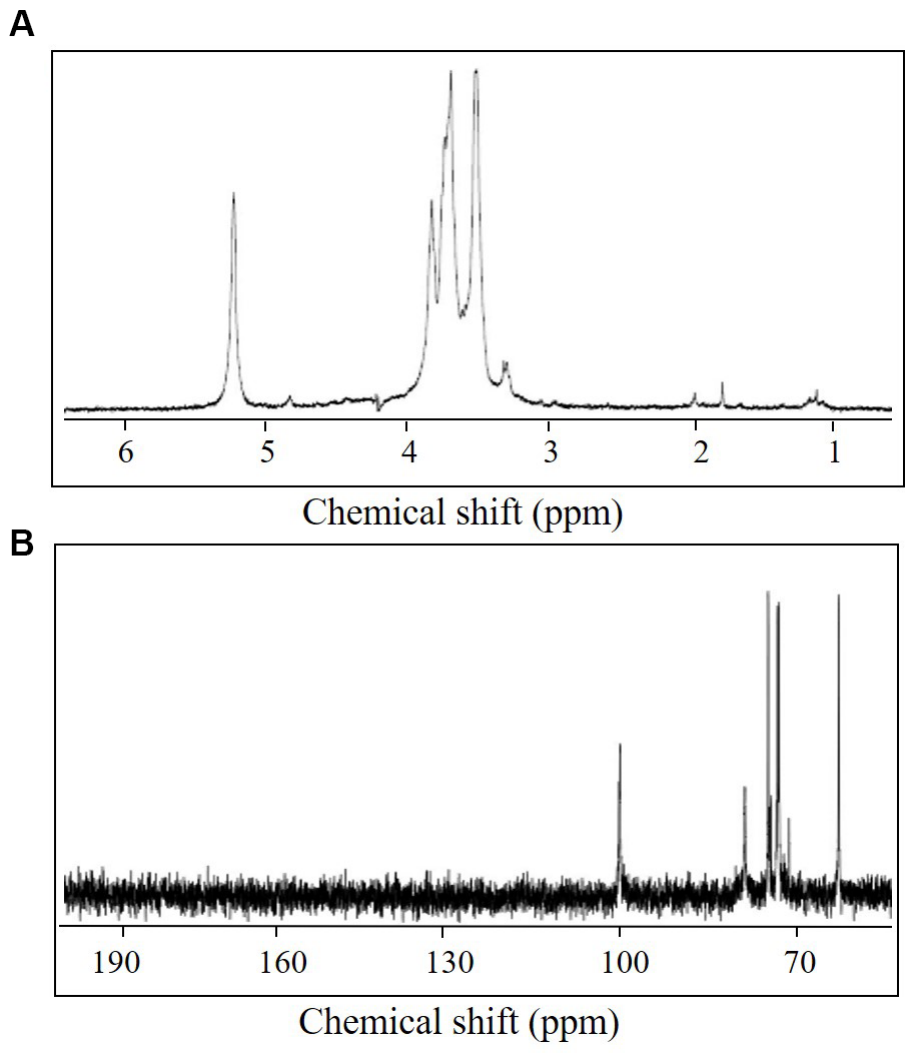
^1^H nuclear magnetic resonance (NMR) spectrum **(A)** and ^13^C NMR spectrum **(B)** of CA42 extracellular polysaccharide, recorded in D_2_O at 70°C. ppm, parts per million.

One resonance signal was observed at δ 5.25 and δ 100.6 in the anomeric regions of the ^1^H and ^13^C NMR spectra, respectively (Figure 3). These anomeric signals were assigned to the α configuration based on the observed chemical shift values (Bubb, 2003). In addition, the ^13^C NMR spectrum showed one signal at δ 61.5 (C-6) and eight at δ 70–79 (C-2–C-5). The ^1^H NMR spectrum displayed several signals at δ 3.31–3.84 (H-2–H-6). The ^1^H chemical shifts of the CA42 EPS were assigned using two-dimensional total correlation spectroscopy (TOCSY) and two-dimensional double quantum filtered-correlation spectroscopy (DQF-COSY) experiments and showed two spin systems (Table 1). The ^13^C chemical shifts of CA42 EPS were assigned using HSQC and HMBC (Table 1). The results of the NMR analyses clearly showed that the CA42 EPS mainly consisted of α-1,4-linked residues of d-glucose.

**Table 1:** ^1^H and ^13^C NMR chemical shifts (ppm) of CA42 EPS recorded in D_2_O at 70°C.

Glycosyl residue	H-1C-1	H-2 C-2	H-3C-3	H-4C-4	H-5C-5	H-6a C-6	H-6b
→4)-α-d-Glc*p*-(1→	5.25 100.6	3.53 72.4	3.84 74.1	3.52 78.3	3.72 72.2	3.69 61.5	3.78
α-d-Glc*p*-(1→	5.25 100.6	3.45 72.7	3.59 73.9	3.31 70.4	3.60 73.6	3.63 61.5	3.73

Next, the CA42 EPS was methylated, and the derived alditol acetates were analyzed using GLC-MS (Table 2). Three peaks were observed, which were identified as 1,5-di-*O*-acetyl-2,3,4,6-tri-*O*-methyl-d-glucitol; 1,4,5-tri-*O*-acetyl-2,3,6-tri-*O*-methyl-d-glucitol; and 1,4,5,6-tetra-*O*-acetyl-2,3-di-*O*-methyl-d-glucitol. The presence of 1,4,5,6-tetra-*O*-acetyl-2,3-di-*O*-methyl-d-glucitol indicated that CA42 EPS is branched at C-6 of the d-glucose residues.

**Table 2:** Methylation analysis of CA42 EPS.

Derivatives	Structural feature	Molar ratio
1,5-Di-*O*-acetyl-2,3,4,6-tri-*O*-methyl-d-glucitol	Glc*p*-(1→	1.2
1,4,5-Tri-*O*-acetyl-2,3,6-tri-*O*-methyl-d-glucitol	→4)-Glc*p*-(1→	12.2
1,4,5,6-Tetra-*O*-acetyl-2,3-di-*O*-methyl-d-glucitol	→4,6)-Glc*p*-(1→	1.0

**Figure 4:**
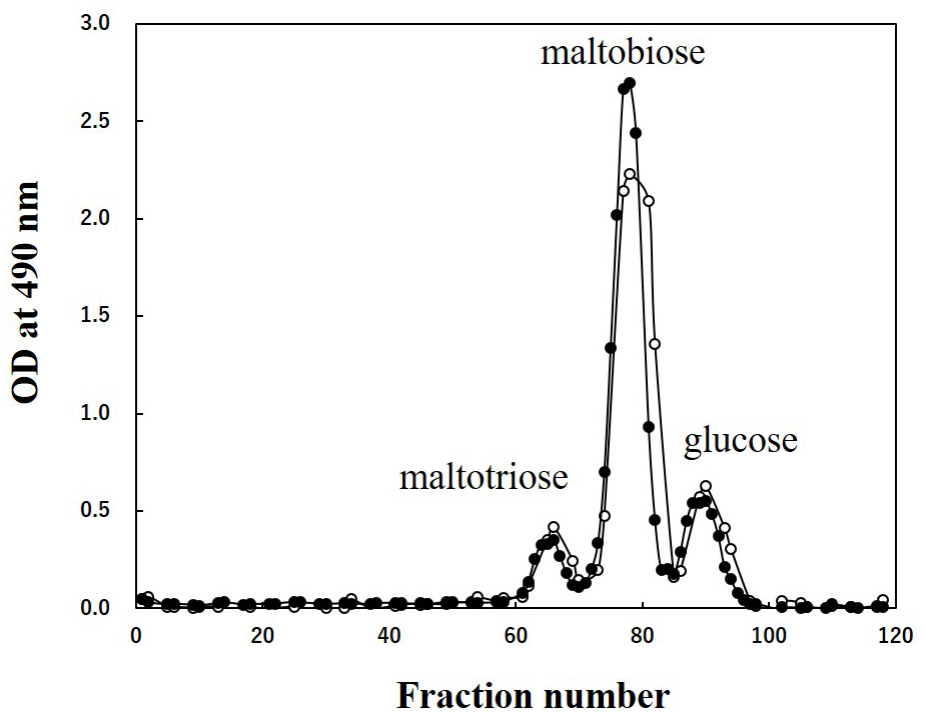
Results of gel filtration column chromatography of CA42 extracellular polysaccharide (EPS) and oyster glycogen treated with pullulanase. Oligosaccharides were applied to the Bio-Gel P-2 gel filtration column (900 mm × 15 mm ϕ), and 0.2 M acetic acid was used as the eluate. Glucose, maltobiose, and maltotriose were used as size markers. Closed circles, CA42 EPS; opened circles, oyster glycogen; OD, optical density.

**Figure 5:**
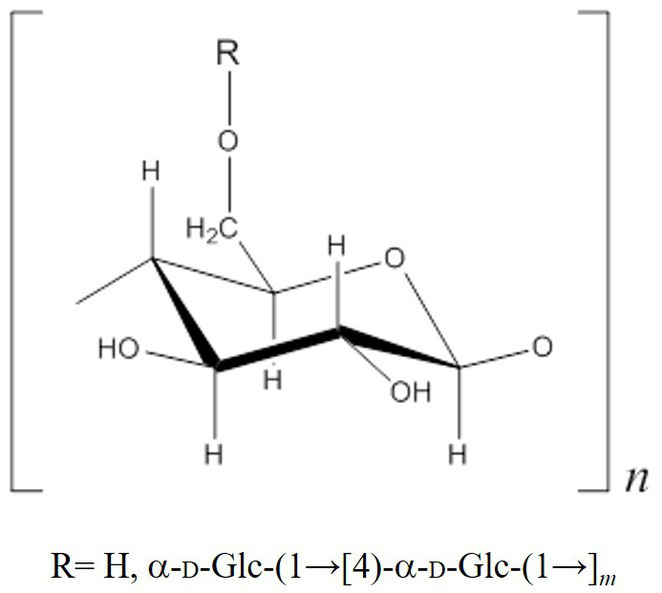
Repeating unit structure of the CA42 extracellular polysaccharide.

CA42 EPS was digested by pullulanase, specifically degrading α-1,6-glucosidic bonds at the branching point of glycogen, and was fractionated using gel filtration column chromatography. Glucose, maltobiose, and maltotriose were detected using the phenol–H_2_SO_4_ method (Figure 4). When oyster glycogen was treated using the same procedure, almost the same elution profile was obtained (Figure 4). These results indicate that CA42 EPS has a side-chain structure similar to that of oyster glycogen. In summary, CA42 EPS is glycogen-like polysaccharide consisting of a repeating unit (Figure 5).

### 3.3. Adsorption of aluminum ion by CA42 EPS

**Figure 6:**
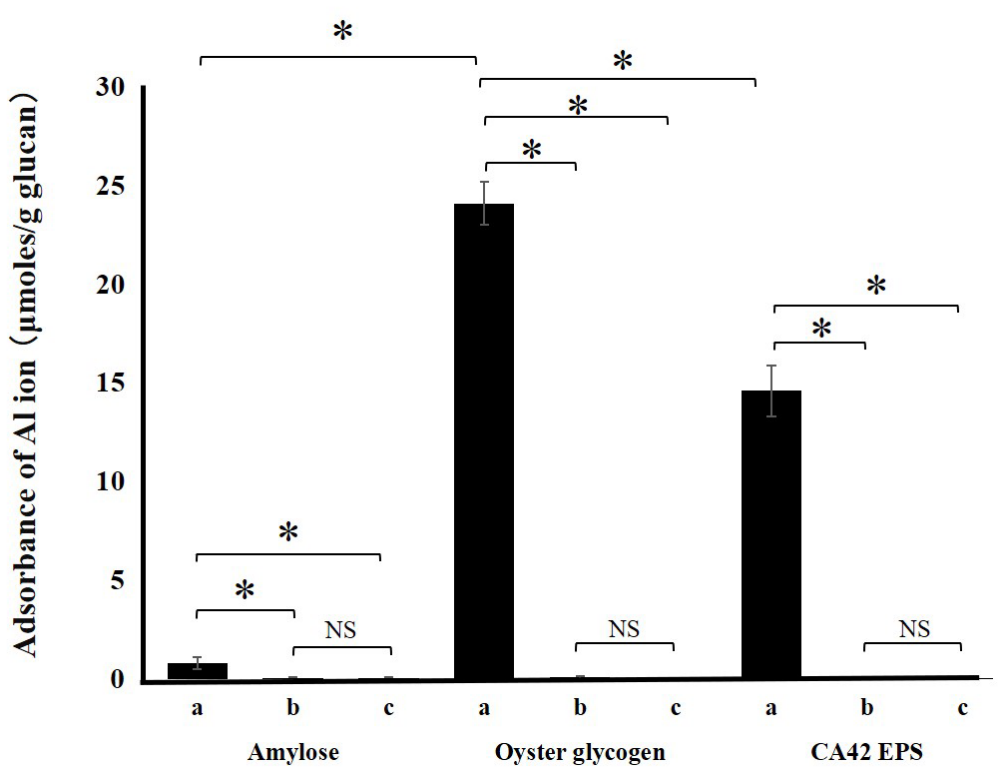
Adsorption of aluminum ion by CA42 extracellular polysaccharides (EPS) and standard glucans. Adsorbed ammonium ion in μmol was divided by the samples in grams. (a), native sample; (b), α-amylase-treated sample; (c), pullulanase-treated sample. *p < 0.01; NS, not significant.

To analyze the role of CA42 EPS in aluminum tolerance, we examined whether the polysaccharide adsorbed aluminum ions (Figure 6). CA42 EPS adsorbed 14.2 ± 0.2 μmol/g (dry weight) aluminum ion at a low pH. Commercially available oyster glycogen also adsorbed 22.8 ± 0.1 μmol/g (dry weight) aluminum ion, while amylose showed little to no adsorption (0.5 ± 0.4 μmol/g dry weight). In addition, enzymatic treatment with either α-amylase or pullulanase completely attenuated the aluminum ion adsorption activity of CA42 EPS, as well as that of oyster glycogen. These results suggest that the glycogen-adsorbed aluminum ion and its branching structure play important roles in the aluminum tolerance of CA42.

### 3.4. Pullulanase treatment attenuated the aluminum tolerance of CA42 cells

**Figure 7:**
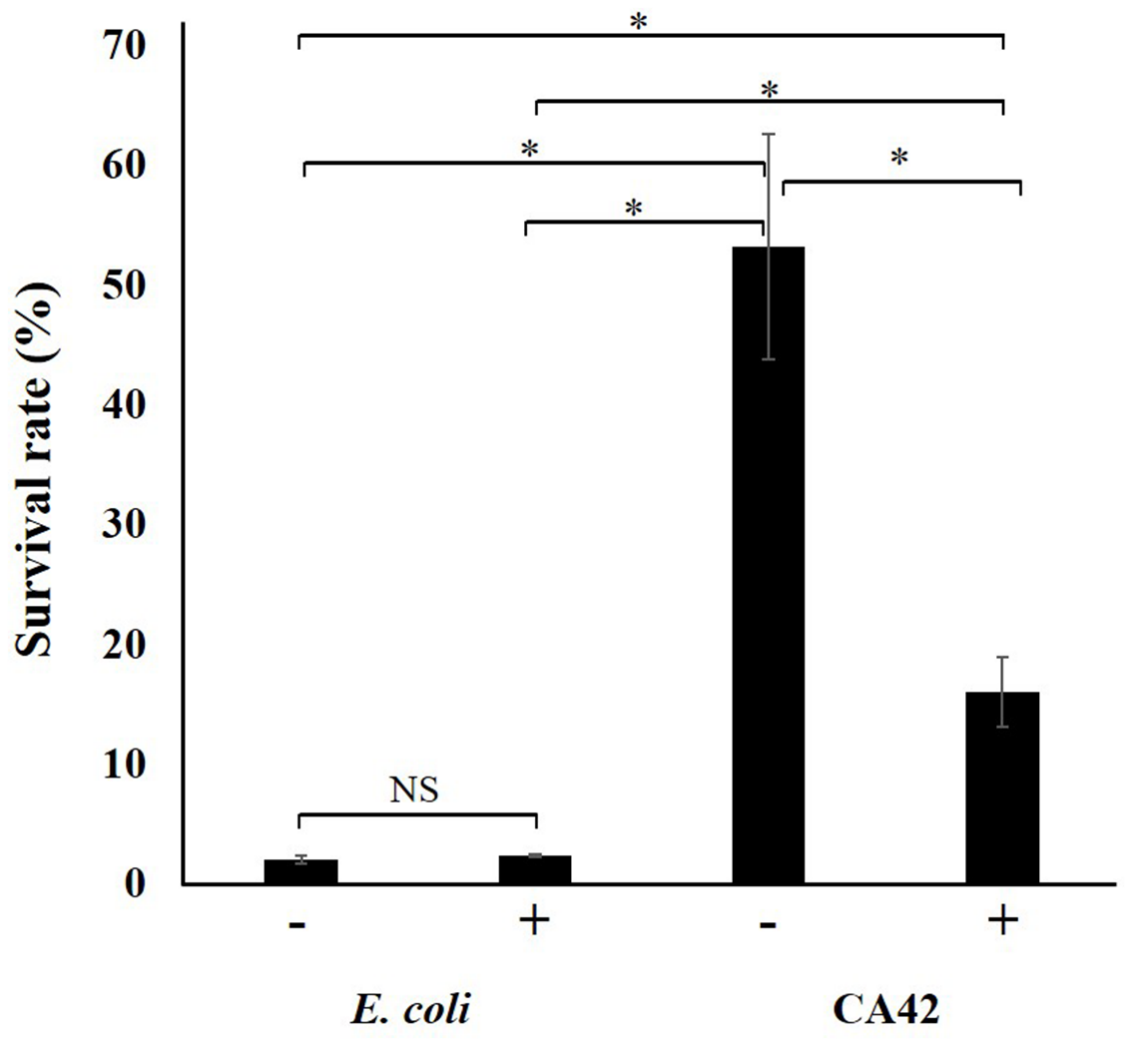
Effect of pullulanase treatment on the aluminum tolerance of CA42. The survival rate of each strain was compared to that of cells not exposed to aluminum ions. *E. coli* was used as the control to evaluate the aluminum toxicity. −, no treatment; +, pullulanase treatment. *p < 0.01; NS, not significant.

Since pullulanase treatment attenuated the aluminum ion-adsorption activity of CA42 EPS, we validated the effect of pullulanase treatment on the aluminum tolerance of CA42 cells. *E. coli* was used to evaluate the aluminum toxicity. The survival rate of each strain was compared with that in the absence of aluminum. The survival rate of *E. coli* was approximately 2%, regardless of pullulanase treatment. CA42 showed 53% survival in the presence of aluminum and 16% survival after pullulanase treatment (Figure 7). These results suggest that CA42 EPS is mainly involved in the aluminum tolerance of *Pullulanibacillus* sp. CA42.

## 4. Discussion

In the present study, we aimed to characterize the novel aluminum-tolerant strain CA42, isolated from AASS, which allows *Oryza sativa* to grow in the presence of 300 μM AlCl_3_ at pH 3.5. The strain was identified as *Pullulanibacillus* sp. CA42, and this is the first strain belonging to the genus *Pullulanibacillus* that showed aluminum tolerance. CA42 produced a large amount of EPS, a glycogen-like α-glucan, that adsorbs aluminum ions. To the best of our knowledge, this study is the first to characterize EPS produced by a strain belonging to the genus *Pullulanibacillus*. The branching structure of CA42 EPS plays an important role in its aluminum adsorption activity. This study is the first report of glycogen-like EPS that adsorb aluminum ions. Furthermore, aluminum tolerance of CA42 was attenuated via pullulanase treatment. Our findings suggest that CA42 can act as a plant growth-promoting bacterium in acidic soils, and that CA42 EPS may protect plants growing in AASS. Currently, we are conducting cloning experiments using CA42 EPS synthetic genes to construct EPS-deficient mutants. With these knockout mutants, we intend to clarify the roles of CA42 EPS in aluminum tolerance and plant growth promotion. The mechanism underlying the aluminum adsorption capacity of CA42 EPS and its application as a new aluminum-adsorbing substance that functions in acidic environments should be elucidated in future studies. This study proposes the new mechanism of aluminum tolerance using a bacterial extracellular polysaccharide. The strain isolated in this study may be a potential plant growth-promoting bacterium in actual acid sulfate soils.

## Data availability statement

The datasets presented in this study can be found in online repositories. The names of the repository/repositories and accession number(s) can be found at: https://www.ncbi.nlm.nih.gov/genbank/, AB520692.

## Author contributions

TA and MU contributed equally to the concept, design, and analysis of the data, as well as the writing of the manuscript. JS and SS contributed to the majority of experiments. TY and YM contributed to the structural polysaccharide analysis. All authors contributed to the article and approved the submitted version.

## Funding

This work was partially supported by the MEXT KAKENHI (grant numbers 20 K05735 and 21 K12298) and Nihon University College of Bioresource Sciences Research Grants for 2018, 2019, and 2020.

## Conflict of interest

The authors declare that the research was conducted in the absence of any commercial or financial relationships that could be construed as a potential conflict of interest.

## Publisher’s note

All claims expressed in this article are solely those of the authors and do not necessarily represent those of their affiliated organizations, or those of the publisher, the editors and the reviewers. Any product that may be evaluated in this article, or claim that may be made by its manufacturer, is not guaranteed or endorsed by the publisher.

## Footnotes

^1^ https://www.ezbiocloud.net/
